# Design and Characterization of Poly(ethylene oxide)-Based Multifunctional Composites with Succinonitrile Fillers for Ambient-Temperature Structural Sodium-Ion Batteries

**DOI:** 10.3390/polym16192806

**Published:** 2024-10-03

**Authors:** Vasan Iyer, Jan Petersen, Sebastian Geier, Peter Wierach

**Affiliations:** 1 Cluster of Excellence SE2A—Sustainable and Energy-Efficient Aviation, Technische Universität Braunschweig, 38108 Braunschweig, Germany; jan.petersen@dlr.de (J.P.); sebastian.geier@dlr.de (S.G.); peter.wierach@dlr.de (P.W.); 2 Department of Multifunctional Materials, German Aerospace Center (DLR), Institute of Lightweight Systems, Lilienthalplatz 7, 38108 Braunschweig, Germany; 3 Institute of Polymer Materials and Plastics Engineering, Technische Universität Clausthal, Agricolastrasse 6, 38678 Clausthal-Zellerfeld, Germany

**Keywords:** structural sodium batteries, structural energy storage, multifunctional materials, fiber-reinforced composites, plasticizers, carbon-fiber electrode

## Abstract

A new approach to developing structural sodium batteries capable of operating in ambient-temperature conditions has been successfully achieved. The developed multifunctional structural electrolyte (SE) using poly(ethylene oxide) (PEO) as a matrix integrated with succinonitrile (SN) plasticizers and glass-fiber (GF) reinforcements identified as GF_PEO-SN-NaClO_4_ showed a tensile strength of 32.1 MPa and an ionic conductivity of 1.01 × 10^−4^ S cm^−1^ at room temperature. It displayed a wide electrochemical stability window of 0 to 4.9 V and a high sodium-ion transference number of 0.51 at room temperature. The structural electrode (CF|SE) was fabricated by pressing the structural electrolyte with carbon fibers (CFs), and it showed a tensile strength of 72.3 MPa. The fabricated structural battery half-cell (CF||SE||Na) demonstrated good cycling stability and an energy density of 14.2 Wh kg^−1^, and it retained 80% capacity at the end of the 200th cycle. The cycled electrodes were observed using scanning electron microscopy, which revealed small dendrite formation and dense albeit uniform deposition of the sodium metal, helping to avoid a short-circuit of the cell and providing more cycling stability. The developed multifunctional matrix composites demonstrate promising potential for developing ambient-temperature sodium structural batteries.

## 1. Introduction

The transportation sector, which is largely reliant on fossil fuels for energy, results in large greenhouse gas emissions that contribute to climate change effects. These effects could be mitigated by transitioning to e-mobility. It is necessary to achieve this transition in the European Union (EU) by 2035, according to the proposed Green Deal [1]. Though the automobile sector is in the phase of transition to electric vehicles from conventional fossil fuel-based vehicles, the aviation sector has much catching up to do. The implementation plan to achieve zero emissions in the aviation sector has been put forward, and the deadline is set for 2050 [2,3].

The current key technology for energy storage in the e-mobility sector is mostly based on Li-ion batteries. As the demand for electric vehicle range increases, it leads to an increase in the battery mass to compensate for the increased energy density requirements. As the vehicle structure and battery remain separated, it contributes to an overall increase in the vehicle mass and reduced energy efficiency. For example, the total battery pack mass for a standard-range (54 kWh version) Tesla Model 3 is 324 kg, or 18.4% of the car mass, whereas, for the long-range model (75 kWh version), the battery mass is increased to 480 kg, or 26.3% of the mass of the car [4]. A possible solution to mitigate such a problem is to integrate the battery within the structure, i.e., to view the battery as a function rather than as a separate component [5]. Such a multifunctional structure with integrated energy storage capability is called a structural battery, and it has the potential to provide massless energy storage, high performance, high overall efficiency, and increased vehicle utility space [6].

The integration of batteries within the structure can be achieved in varied degrees of integration (DOI), with DOI level 0 being the current-day electric vehicle architecture, where the batteries and structure are totally separated. In DOI level I, the batteries are integrated into the empty spaces within the structure, and in DOI level II, the thin film battery is attached to the surface of the structure [7,8,9]. The complexity of the structural battery design increases with higher DOI levels (III and IV), where true multifunctionality is achieved, in which the battery is now seen as a function and cannot be separated from the structure [10,11]. In higher DOI structural batteries, each component of the battery plays multiple roles and contributes both to structural strength and ion storage and provides a pathway for the movement of ions. The main challenges in achieving higher DOI designs include the optimization of both the mechanical and the electrochemical properties of the battery components. Currently, the reported energy density values of higher DOI structural batteries are comparatively lower than conventional batteries by one or two orders of magnitude, and typical values lie in the range of 1–50 Wh kg^−1^ [12]. The ultimate goal of structural battery research is to achieve both high DOI and energy density to provide massless storage and overall high energy efficiency.

Previous studies have used carbon-fiber (CF) electrodes in developing multifunctional energy storage materials [12,13,14,15]. CF is interesting as it possesses both mechanical and electrochemical properties and is also an important component for manufacturing composites for transport applications [16,17]. In the state-of-the-art DOI III structural battery architecture, carbon fibers are used as they contribute to the structural strength as reinforcements, act as electrodes, and store ions. Previous studies mainly focused on structural battery designs based on lithium-ion intercalation into CF and investigated bi-continuous phase-type electrolytes and all solid-state-type electrolytes with glass-fiber separators as the multifunctional matrix material [14,15,16,17,18,19]. These materials contribute to structural strength as well as facilitate ion transport.

In contrast, there are few structural battery architectures based on sodium-ion intercalation into CF, thus requiring further investigation. This study addresses this gap and investigates structural battery designs based on sodium-ion intercalation using all solid-state composite-type electrolytes as multifunctional matrix materials. The most important consideration for designing sodium structural batteries lies in the way in which the sodium-ion intercalates into carbon fibers. Lithium ion can intercalate in between graphitic layers, edges, and lattice surfaces, whereas sodium ion mainly intercalates onto lattice surfaces and in pores/defect sites of the carbon-fiber microstructure. Hard carbon possesses such characteristic pores/defect sites that favor sodium-ion intercalation [12,17,20]. The investigation of intermediate modulus (IM)-type carbon fibers reveals that they possess a microstructure that resembles hard carbon, and hence, IM-type carbon fiber is used to construct electrodes for sodium-ion intercalation in the present study [17,21].

The choice of polymer matrix plays a crucial role in the design of a multifunctional matrix composite for structural batteries. In the present study, high-molecular-weight poly(ethylene oxide) or PEO is chosen as a matrix for the preparation of solid polymer electrolytes (SPEs). PEO has the unique advantages of good mechanical properties, high safety, and a low glass transition temperature, and it offers good thermal, electrochemical, and interfacial stability due to the high solubility of sodium salts [22,23]. Even though PEO possesses many good characteristics, the room-temperature ionic conductivity of the PEO-based electrolytes with alkali metal salts is significantly low. This is mainly due to the crystalline nature of PEO [22,23]. The addition of inorganic active fillers has been proven to reduce the crystalline nature of PEO and enhance ion transport [24,25].

In a previous study of sodium-ion-based structural batteries involving the fabrication of multifunctional composites using inorganic ceramic NZSP (Na_3_Zr_2_Si_2_PO_12_) nanoparticle fillers, the battery showed good cycling stability and retained 80% capacity at the end of 225 cycles. However, it operated at high temperatures [12]. Therefore, a strategy is needed for designing a sodium structural battery for ambient-temperature operation. Previous studies reported that the plastic crystalline phase of succinonitrile (SN) showed high solubility for various salts due to its high polarity. The investigated electrolytes displayed high ionic conductivities in a wide range of temperatures, with values reaching 3 × 10^−3^ cm^−1^ at 25 °C, which are two to three orders of magnitude higher than other polymer-based matrices at 25 °C [26]. Furthermore, studies involving PEO-based matrices with SN fillers also reported high ionic conductivities at room temperature [27,28,29]. These results give the motivation for the present study to fabricate multifunctional composites using SN as plasticizers.

This study reports the first-of-its-kind multifunctional composites fabricated using all solid-state PEO-based electrolytes with succinonitrile (SN) plasticizers for ambient-temperature sodium structural batteries. The composites are investigated for their multifunctional performance. The structural electrolyte was prepared using a two-step approach, where first, the polymer membrane or the composite solid electrolyte was prepared using a solution-casting technique, and then it was reinforced with glass fibers to manufacture the structural electrolyte (GF_PEO-SN-NaClO_4_). The structural electrolyte is a multifunctional composite matrix material with the tasks of transferring mechanical loads and transporting sodium ions. The glass-fiber reinforcement is done to further improve the mechanical characteristics of the structural electrolyte, and it displayed a tensile strength of 32.1 MPa and ionic conductivity of 1.01 × 10^−4^ S cm^−1^ at room temperature. The structural electrode component was fabricated by laminating the prepared structural electrolyte with intermediate modulus (IM) type carbon fibers, which showed a tensile strength of 72.3 MPa. The fabricated structural battery half-cell (CF || GF_PEO-SN–NaClO_4_ || Na) provided a typical energy density of 14.2 Wh kg^−1^ at 0.1 C rate, and the cycling performance tested at 0.9 C rate showed 80% capacity retention after 200 cycles.

## 2. Experimental Section

### 2.1. Materials and Methods

Chemicals and materials for the synthesis of structural electrolytes include poly (ethylene oxide) (PEO (molecular weight (M_w_) of 10^6^ g mol^−1^), Sigma-Aldrich, Steinheim, Germany), anhydrous acetonitrile(ACN with 99% purity, Sigma-Aldrich, Steinheim, Germany), Succinonitrile (C_2_H_4_(CN)_2_, Sigma-Aldrich, Steinheim, Germany), sodium perchlorate salt (NaClO_4_, Sigma-Aldrich, Steinheim, Germany), glass-fiber woven fabric with an aerial weight of 163 g m^−2^ (Aero, R&G Faserverbundwerkstoffe GmbH, Waldenbuch, Germany), spread tow carbon fibers (CF) Tenax IMS65 24k tows with aerial weight of 55 g m^−2^ was acquired from R&G Faserverbundwerkstoffe GmbH, Waldenbuch, Germany for the fabrication of the structural electrode. Sodium (Na) metal sticks for the preparation of sodium foils were purchased from Alfa Aesar, Karlsruhe, Germany. Siliconized (double-sided) papers for preparing structural electrolytes and electrodes using a press were obtained as a gift from Laufenberg GmbH, Krefeld, Germany.

### 2.2. Material Characterization and Analysis

The SEM (scanning electron microscopy using FEI, Thermo Fisher Scientific Inc., Dreieich, Germany) and energy dispersive X-ray analysis (EDAX) techniques were used for studying the morphology and characterizing the fabricated structural electrolyte and electrode.

### 2.3. Preparation of Polymer Membranes and Structural Electrolytes

**Figure 1:**
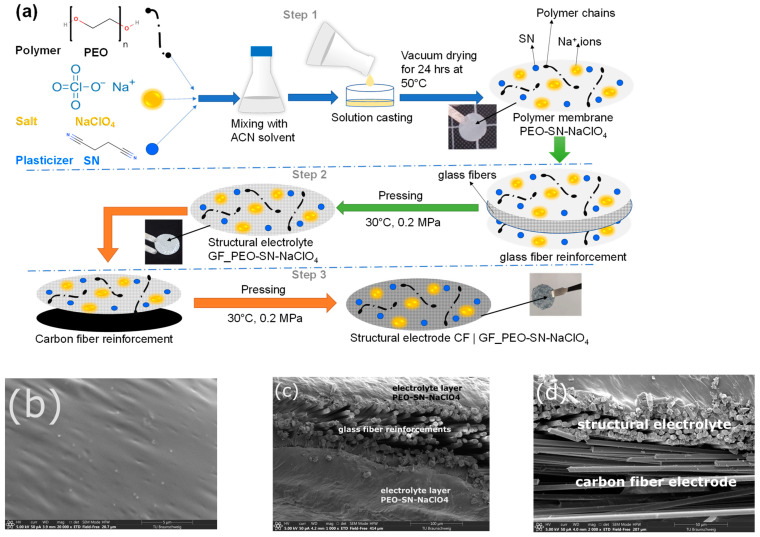

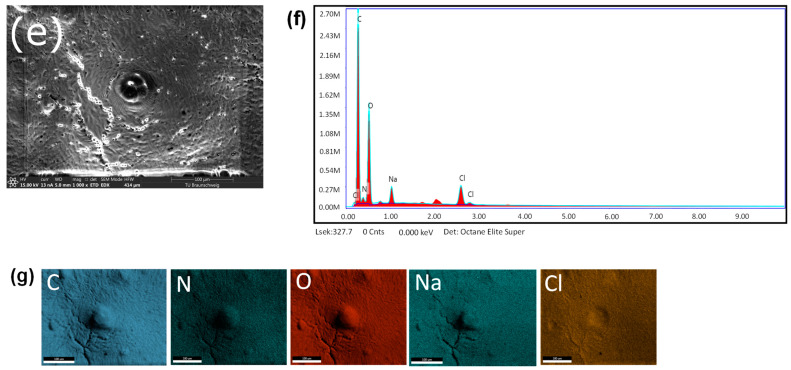
Multifunctional composite preparation and characterization: (**a**) schematic drawing showing the manufacturing process of the structural electrolyte and structural electrode. SEM images: (**b**) Polymer membrane PEO-SN–NaClO_4_. (**c**) cross-sectional view of the structural electrolyte GF_PEO-SN–NaClO_4_. (**d**) cross-sectional view of the structural electrolyte CF || GF_PEO-SN–NaClO_4_. (**e**) structural electrolyte surface after EDAX. (**f**) EDAX pattern for the structural electrolyte GF_PEO-SN–NaClO_4_. (**g**) SEM–EDAX element mapping for the structural electrolyte.

The polymer membranes were prepared as described in the schematic drawing shown in Figure 1a, and Figure S1 shows the complete process flow for the fabrication of structural electrolytes. In the first step, the thin electrolyte membranes were prepared using the solution-casting technique. Initially calculated amounts of PEO and NaClO_4_ were added to the (ACN) organic solvent in a sealed container and stirred for 4 h. The ratio of ethylene oxide (-CH_2_-CH_2_-O-) to sodium ion (EO:Na^+^) is maintained at 15:1. Then various amounts (30–40% by weight) of succinonitrile (SN) were added to the mixture, and it was sonicated (using a Branson 250 Digital Sonifier, Branson, New Hampshire, USA) for 30 min and further stirred for 12 h until a homogenous mixture is obtained. The solution is then cast into a PTFE evaporation dish and set aside in the fume hood for 10 h until a layer with a uniform surface and thickness is formed. The membranes so obtained are vacuum dried for 24 h at 50 °C to evaporate the ACN solvent completely and to get thin polymer membranes identified as PEO-SN–NaClO_4_ (Figure S2) with thickness measured to be 180 µm. In the second step, the glass fibers were reinforced and sandwiched between the two thin polymer membranes (Figure 1a (step 2)) utilizing a press (Collins P500S press, COLLIN Lab & Pilot Solutions GmbH, Maitenbeth, Germany) to fabricate the structural electrolyte with a thickness of 360 µm. The pressing was carried out for 25 min at room temperature and 0.2 MPa pressure. The adhesive nature of the thin polymer membrane helps in achieving good integration with glass fibers. The glass-fiber-reinforced structural electrolyte is identified as GF_PEO-SN-NaClO_4_ (Figure S2).

### 2.4. Preparation of Structural Electrode

The structural electrodes were prepared by placing the structural electrolyte over a layer of spread tow carbon fiber in between the siliconized papers to prevent sticking to the press plates. It was then pressed at room temperature with 0.2 MPa pressure using a Collins P500S press for 25 min, as depicted in Figure 1a (step 3). The adhesive nature of the prepared structural electrolytes allowed seamless integration and bonding with the carbon-fiber layer. Figure S3 shows both the top and bottom surfaces of the prepared structural electrode.

### 2.5. Electrochemical Characterization and Analysis

The sodium-ion conductivities of the prepared electrolytes were evaluated using electrochemical impedance spectroscopy (EIS) using the four-point electrode cell technique. An RHD measuring cell TSC Battery (manufactured by RHD Instruments GmbH & Co. KG, Darmstadt, Germany) with stainless-steel blocking electrodes was used to record the EIS spectra. The electrolytes, with a size of 8 mm diameter, were used to make the EIS recordings in the set frequency ranges from 1 Hz to 1 MHz, and the sinusoidal perturbation was set to 10 mV in the Zahner potentiostat (Zhennium, Zahner-Elektrik GmbH & Co. KG, Kronach, Germany).

The sodium-ion transference numbers were assessed using a symmetrical cell (Na || electrolyte || Na) prepared in the 2032-coin cell configuration (Figure S4). The AC–DC (alternating current–direct current) polarization technique was used, where initially, a 10 mV AC polarization voltage was applied to the symmetrical cell, and the EIS spectrum was recorded in the frequency range 1 Hz to 1 MHz. From the obtained Nyquist plot, the charge transfer resistance (*R*_0_) was then calculated. A DC polarization voltage of 10 mV is applied, and the chronoamperogram (which records the evolution of current with respect to the applied DC polarization voltage) is observed until the current reaches a constant steady-state value (*I_SS_*). The starting current (*I*_0_) in the current evolution is obtained from the experiment. The EIS spectrum was recorded once again, and the steady-state charge transfer resistance (*R_SS_*) was calculated from the Nyquist plot [30].

The electrochemical stability window (ESW) of the fabricated electrolytes was evaluated using linear sweep voltammetry (LSV) using the prepared cells in asymmetric configuration (SS || Electrolyte || Na) with stainless-steel (SS) electrode and sodium (Na) foil counter electrode (Figure S4). A positive scan from 2.5 V to 6.5 V and a negative scan from 2.5 V to 0 V was conducted with a scan rate of 1 mV s^−1^ at 30 °C.

### 2.6. Mechanical Characterization

The mechanical strength of the multifunctional components (structural electrolyte and structural electrode) was assessed by performing tensile tests with a 5 kN load cell (using Zwick-Roell universal testing machine, UTM). A high-quality 3D industrial camera (ZEISS ARAMIS 3D, Carl Zeiss IQS GmbH, Oberkochen, Germany) setup was used to capture precise strain measurements. To pixelate the samples with a stochastic black-and-white pixel distribution, paint in a speckle pattern was used to cover the surface of the sample, which was then exposed to the camera. This helps in more accurate elongation measurements. All samples were prepared according to the ASTM D-638 Type V standard, and the loading rate of the cell was set at 2 mm min^−1^.

### 2.7. Structural Battery Half-Cell Assembly and Testing

The structural battery performance was evaluated using the 2032-coin cell configuration (Figure S5). The fabricated structural battery (Na||GF_PEO-SN-NaClO_4_||CF) consists of carbon fiber as a positive electrode and sodium foil as the counter electrode. For the construction of the cell, a structural electrode with an 8 mm diameter was used, and a disc-shaped sodium (Na) foil measuring 13 mm in diameter was positioned on top of the electrolyte surface. The sodium foil was initially pressed firmly against the stainless-steel (SS) spacer surface. A second SS spacer was placed below the structural electrode (carbon-fiber surface) to serve as a current collector. The coin cell assembly was conducted inside the glove box (in a strict protective environment with humidity of 0 ppm and oxygen of 0 ppm) using the MTI MSK-160E pressure adjustable electric crimper (manufactured by MTI Corporation, Richmond, CA, USA). The cell was inserted into a Gamry dual 2032-coin cell battery holder (Part number: 992-00159) and was charged–discharged galvanostatically using the potentiostat Reference 3000 (Gamry LCC, Warminster, PA, USA). The capacity of the structural battery was assessed based on the weight of the carbon fiber used.

## 3. Results and Discussion

Figure 1b,c depict the surface and cross-section of the structural electrolyte captured using scanning electron microscopy (SEM). Figure 1b is magnified at 20,000×, while Figure 1c is magnified at 1000×. SEM images were taken with a landing energy of 5000 V. In Figure 1c, the cross-section image shows the glass-fiber reinforcements located between the two electrolyte layers. Figure 1e displays the SEM image of the structural electrolyte post-EDAX analysis (20,000× magnification, 15,000 V Landing energy), while Figure 1f shows the EDAX pattern for the same. The EDAX was performed with the following parameters (kV: 15, Magnification: 876; Angle of view: 35.6; Filter time (µs): 0.9; Resolution: (eV) 126.9). Figure 1g displays the distribution of elements, with both the EDAX pattern and SEM–EDAX mapping confirming the presence of SN plasticizers and sodium perchlorate salt in the structural electrolyte. Figure 1d displays the structural electrode cross-section through SEM imaging at a magnification of 2000, revealing the structural electrolyte with embedded glass fiber above and the carbon-fiber electrode below.

### 3.1. Electrochemical Characterization of Structural Electrolyte

Some key factors of the multifunctional structural electrolyte to be considered for structural battery application include ionic conductivity, ion transference number, the electrochemical stability window, cycling stability, and mechanical property. The prepared electrolytes are initially evaluated for their sodium-ion conductivities using the electrochemical impedance spectroscopy (EIS) technique, as explained in Section 2.

**Figure 2:**
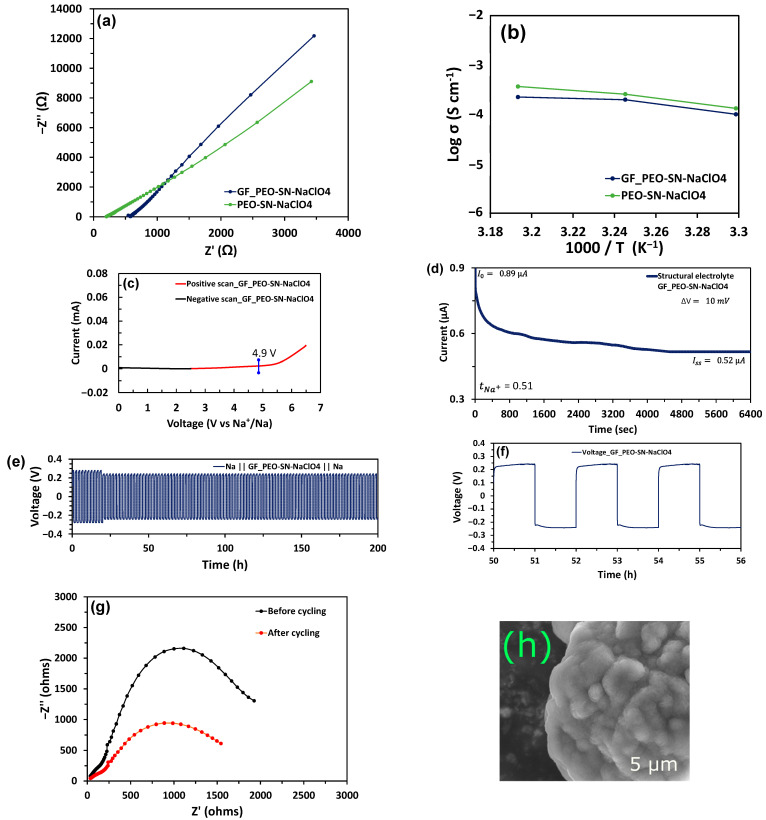
Electrochemical characterization: (**a**) EIS of structural electrolyte (GF_PEO-SN-NaClO_4_) and PEO-SN-NaClO4 at room temperature. (**b**) Arrhenius plots of electrolytes from 30 °C to 45 °C. (**c**) Linear sweep voltammetry (LSV) curve of Structural electrolyte GF_PEO-SN-NaClO_4_. (**d**) Chronoamperogram or current response of Na || GF_PEO-SN-NaClO_4_ || Na symmetrical cell to applied DC polarization. (**e**) Galvanostatic cycling of symmetrical cells Na||GF_PEO-SN-NaClO_4_||Na for a current density of 0.1 mA cm^−2^. (**f**) Typical voltage profile of the Na||GF_PEO-SN–NaClO_4_||Na cell from 50 to 56 h. (**g**) EIS of Na || GF_PEO-SN-NaClO_4_ || Na symmetrical cell before and after cycling. (**h**) SEM image of the Na electrode for the Na||GF_PEO-SN-NaClO_4_||Na cell after cycling at 5 μm range.

The ionic conductivities of the electrolytes are evaluated using the bulk resistance (*R_b_*) determined from the Nyquist plots (Figure 2a and Figure S6). The value of *R_b_* is determined by where the Nyquist plot intercepts the real axis and is also evaluated using the EIS equivalent circuit (as shown in Figure S7). The calculation for ionic conductivity is made using Equation (1)

(1) $$\sigma =\frac{t}{{AR}_{b}}$$

where *t* is the thickness of the electrolyte, and *A* is the geometrical contact area of the electrolyte with the stainless-steel electrodes. The polymer composite electrolytes are prepared using various proportions of PEO and the plasticizer succinonitrile (SN). The electrolyte, composed of 55% PEO and 35% SN by weight, exhibited the highest ionic conductivity of 1.32 × 10^−4^ S cm^−1^ at room temperature, along with good structural integrity. Hence, this is utilized for the fabrication of structural electrolytes. Though the electrolyte composition with 50% PEO and 40% SN showed maximum ionic conductivity of 3.09 × 10^−4^ S cm^−1^ at room temperature, the texture is too soft and not suitable for structural electrolyte preparation. Table S1 summarizes the room-temperature ionic conductivities of investigated electrolyte compositions.

Figure 2a shows the Nyquist plots of the prepared structural electrolyte GF_PEO-SN–NaClO_4_ and polymer membrane (with 55%PEO-35%SN) designated as PN-SN-NaClO_4_. The structural electrolyte showed an ionic conductivity of 1.01 × 10^−4^ S cm^−1^ at room temperature, suitable for ambient-temperature battery operation. However, it is lower than PEO-SN-NaClO_4_ due to the presence of an insulative glass-fiber layer. The amorphous nature of the electrolyte helps in maintaining high ionic conductivity even after glass-fiber reinforcements. Figure 2b shows the Arrhenius profile of the electrolytes, and the comparison of ionic conductivities of various prepared electrolytes is summarized in Figure S8.

The linear sweep voltammetry (LSV) measurements were conducted using asymmetric cells (SS || GF_PEO-SN–NaClO_4_ || Na) to evaluate the electrochemical stability window (ESW) of the structural electrolyte. The positive scan was conducted from 2.5 V to 6.5 V, while the negative scan ranged from 2.5 V to 0 V with a scan rate of 1 mV s^−1^ at room temperature. The LSV curve for the structural electrolyte is shown in Figure 2c. The structural electrolyte showed a wide ESW of 0 to 4.9 V, beyond which the current steadily increases with each applied millivolt. The ESW was discovered to be greater than the value obtained for pure PEO-NaClO4 (Figure S9), which is between 0 and 3.95 V [12]. This indicates that both the addition of the SN plasticizer and the glass-fiber reinforcements have widened the ESW due to their stabilization effects [31].

The transference ion number of the structural electrolyte (GF_PEO-SN-NaClO_4_) was evaluated using the AC–DC experiments described in Section 2. The chronoamperogram captured data for a 10 mV DC polarization voltage in Figure 2d, indicating values for initial current (*I*_0_) and steady-state current (*I_SS_*). The EIS Nyquist plot (Figure S10) gives the initial charge transfer resistance (*R*_0_) and the final steady-state resistance (*R_SS_*) before and after applying DC polarization, respectively.

The transference ion number was obtained using Equation (2) [30].

(2) $$t_{{Na}^{+}}=\frac{I_{ss}\left(\Delta V-I_{0}R_{0}\right)}{I_{0}\left(\Delta V-I_{ss}R_{ss}\right)}$$

The structural electrolyte exhibited a sodium-ion transference number of 0.51 at room temperature, comparable to the reported value of 0.48 in the literature [29]. This high value can be attributed to the addition of succinonitrile plasticizers and their ability to provide sodium-ion pathways and to the addition of glass fibers, which augments sodium-ion movement [19].

To assess the performance and cycling stability of the structural electrolyte, the symmetrical cell configuration (Na||GF_PEO-SN-NaClO_4_||Na) was galvanostatically charged and discharged using a current density of 0.1 mA cm^−2^. Figure 2e displays the cycling curves for the symmetrical cell, while Figure 2g shows the EIS Nyquist plots before and after cycling. The cell displayed a high initial charge transfer resistance (R_CT_) value of 2010 Ω, which is evident in the initial voltage rise in the symmetrical cell (Figure 2e). The localized sodium plating and unstable interface account for this early high R_CT_ [32,33]. After approximately 20 h, the cell voltage drops as the interface stability improves, and a structured voltage profile is displayed (as depicted in Figure 2f). The reduced R_CT_ value of 1490 Ω obtained from the Nyquist plot (Figure 2g) indicates the stability of the interface. In total, the cell operated continuously for 200 h, and the voltage profile between 50 and 56 h is displayed in Figure 2f. Figure 2h, with a magnification of 25,000 and a landing energy of 10,000 V, depicts an SEM image of the Na electrode in the Na||GF_PEO-SN-NaClO_4_||Na symmetrical cell after 200 cycles, illustrating compact yet uniform sodium depositions with minor dendrites to prevent cell short-circuiting which is usually caused by larger dendrite formations.

### 3.2. Mechanical Characterization of Multifunctional Composites

The tensile strength of the fabricated composites was determined by conducting mechanical tests on the prepared structural electrolyte and electrode samples using a tensile testing machine, as outlined in Section 2. A ZEISS ARAMIS industrial camera is utilized to acquire accurate strain measurements during the test.

**Figure 3:**
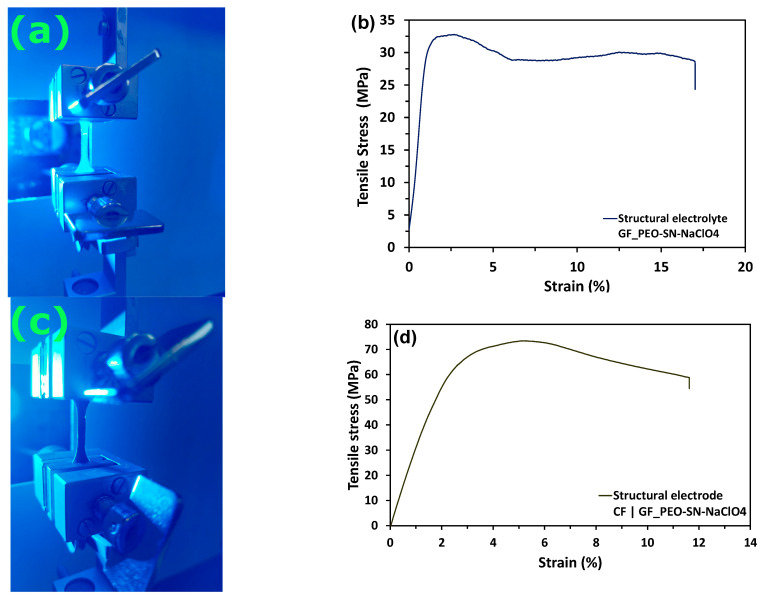
Mechanical characterization: (**a**) Experimental setup for the tensile test of structural electrolyte. (**b**) Typical stress–strain curve of structural electrolyte GF_PEO-SN-NaClO_4_. (**c**) Experimental setup for the tensile test of structural electrodes. (**d**) Typical stress–strain curve of structural electrode CF|| GF_PEO-SN-NaClO_4_.

Figure S11 displays the strain measurements of the samples from the start point to the maximum load point. The structural electrolyte’s tensile strength was assessed using a 5 kN load cell arrangement illustrated in Figure 3a. The stress–strain graph in Figure 3b displays a maximum tensile strength of 32.1 MPa, which is attributed to incorporating glass fibers into the production of the structural electrolyte. The average tensile stress measured in the samples was 29.4 MPa, with a standard deviation of 5.73 MPa, as illustrated in Figure S13. The Young’s modulus is determined to be 1.08 GPa. The anticipated result showed that the tensile stress value is less than the 40.9 MPa reported in the prior study on multifunctional polymer composite with NZSP nanoparticle fillers [12]. The main reason for this is the incorporation of succinonitrile plasticizers, which increase the amorphous nature of the matrix, resulting in a decrease in the overall tensile strength of the multifunctional matrix composite.

The tensile test setup with a 5 kN load cell for the structural electrode is shown in Figure 3c, while the stress–strain curve is shown in Figure 3d. The strain measurements of the structural electrode sample are shown in Figure S12. The structural electrode material exhibited a typical tensile strength of 72.3 MPa. The increase is due to the lamination of carbon fiber onto the structural electrolyte. Figure S13 displays a mean tensile stress of 76.6 MPa with a standard deviation of 9.6 MPa. The calculation of Young’s modulus gives a value of 2.12 GPa. Once again, the structural electrode’s tensile strength is slightly lower at 91.3 MPa than the previous value reported [12]. The primary reason for this is the variation in the types of fillers utilized in these research studies. Figure S14 displays the force–displacement curves for the different structural electrolyte and structural electrode samples that were tested.

### 3.3. Structural Battery Fabrication and Performance

**Figure 4:**
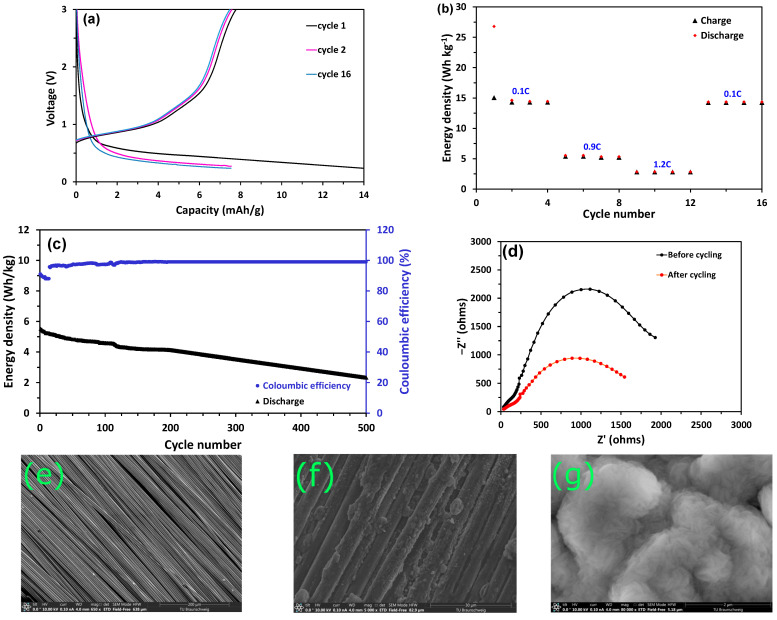
Structural battery performance: (**a**) Charge-discharge profiles of CF || GF_PEO-SN-NaClO_4_ || Na cell for 0.1 C rate. (**b**) Rate capability tests showing specific capacity Vs cycle number of CF || GF_PEO-SN-NaClO_4_ || Na cell at different C rates. (**c**) Stability tests showing charge-discharge energy density and Coulombic efficiency Vs cycle number of CF || GF_PEO-NZSP–NaClO_4_ || Na cell for 0.9C rate. (**d**) EIS of CF || GF_PEO-SN-NaClO_4_ || Na cell before and after cycling. (**e**) SEM image of pure carbon-fiber electrode: (**f**) SEM image of carbon-fiber electrode after cycling. (**g**) SEM image of Na electrode after cycling.

The structural battery was fabricated as described in Section 2 in the 2032-coin cell configuration (Figure S3), with spread tow carbon-fiber (CF) cathode and sodium metal anode (CF || GF_PEO-SN-NaClO_4_ || Na). Figure 4a shows the cell’s charge-discharge profiles at a 0.1 C rate from 0.2 V to 3 V, with a nominal voltage of 1.9 V during discharge. The aerial weight of the carbon fibers (55 g m^−2^) was used for the computation of cell capacities. The cell exhibited a high discharge capacity of 14 mAh g^−1^ in the first cycle because of the stable formation of the solid interface layer (SEI), with a Coulombic efficiency of 58% [34]. From successive cycles, the Coulombic efficiency improved significantly in the range of 96–98%. The typical discharge capacity was found to be 7.5 mAh g^−1^, and the corresponding energy density at 0.1 C rate was estimated to be 14.2 Wh kg^−1^. The energy density of the current work is comparatively lower than our prior study on a PEO-based multifunctional composite containing active NZSP nanoparticle fillers, which achieved an energy density of 23 Wh kg^−1^. This is because NZSP is sodium-rich, and the vacant sites in its solid-state structure aid in the diffusion of ions, contributing to high ionic conductivity and higher energy density [12].

To assess the performance of the cell at different C rates, rate capability tests were conducted. The cell was charged–discharged for 4 cycles at each C rate, and the energy density vs cycle number plot is shown in Figure 4b. For 0.1 C, 0.9 C, and 1.2 C, the cell displayed energy densities of 14.2 Wh kg^−1^, 5.5 Wh kg^−1^, and 2.6 Wh kg^−1^, respectively. The energy density at 0.1 C rate remained fairly constant from the 13th to the 16th cycle.

These values reflect the current trends in DOI III structural battery design, with reported energy densities ranging from 1 to 50 Wh kg^−1^ [12]. Table 1 summarizes and compares the various reported energy densities for DOI III structural batteries with the values reported in this study, while Figure S15 summarizes the energy density versus elastic modulus plot of various DOI III structural battery designs [10,35,36,37,38,39]. The primary difficulty in designing structural batteries is enhancing both their mechanical and electrochemical capabilities. In Figure 4c, the discharge profile for a 0.9 C rate is displayed along with the Coulombic efficiencies measured for each cycle. The cell exhibited good cycling performance and could retain 80% capacity at the end of 200 cycles, and the Coulombic efficiency remained high at 98% consistently after the 12th cycle once the structural electrolyte-carbon-fiber interface was stabilized. The cell capacity degradation plot is shown in Figure S16. The capacity degradation occurs because of the alteration in charge transfer resistance (R_CT_), as seen in the EIS Nyquist plot in Figure 4d. At the start of the initial cycle, the resistance of the R_CT_ was 980 Ω, and by the conclusion of the 200th cycle, it had risen to 2200 Ω, which can be attributed to the formation of sodium dendrites. Figure 4e (Magnification: 650; Landing Energy: 10,000 V) and Figure 4f (Magnification: 5000; Landing Energy: 10,000 V) compare the SEM images of the carbon-fiber electrodes before and after cycling, respectively, while Figure 4g (Magnification: 80,000; Landing Energy: 10,000 V) shows the Na electrodes after cycling. The formation of dendrites is evident from these images. It is small with dense but uniform Na deposition (as shown in Figure 4g). This helps in avoiding short circuits of the cell and enhances cycling stability. The cycling stability of the structural battery could be further credited to the formation of a stable solid electrolyte interphase (SEI) layer. It is expected that the cell energy density could be further boosted by the treatment of carbon fibers and further stacking of cells, which is essential for high energy density structural battery architectures, along with using less resistive materials instead of glass fibers. Further steps in realizing the full structural battery cell include the usage of suitable carbon fibers coated with sodium-abundant cathode material instead of Na metal as an active material providing a sodium-ion source. In this case, the carbon fibers will act both as electrodes and as current collectors. Such an architecture is expected to provide pathways to design structural batteries capable of operating in ambient-temperature conditions. Since succinonitrile plasticizers are used in the present multifunctional composite system with a melting point of 58 °C, it is necessary to adapt low-temperature curing resin systems for the manufacture of multifunctional structural components using these multifunctional composites [40].

## 4. Conclusions

This work developed and characterized multifunctional composites suitable for designing ambient-temperature sodium-ion structural batteries. Some of the key findings are summarized below:

- The structural electrolyte (GF_PEO-SN-NaClO_4_) was fabricated using a poly(ethylene oxide) (PEO)-based matrix with succinonitrile fillers, and it was reinforced with glass fibers. This is important to achieve high tensile strength electrolytes suitable for designing structural batteries.
- The mechano-electrochemical characterization of the structural electrolyte showed a tensile strength of 32.1 MPa and an ionic conductivity of 1.01 × 10^−4^ S cm^−1^ at room temperature. It displayed a wide electrochemical stability window of 0 to 4.9 V and a high sodium-ion transference number of 0.51. The multifunctional performance shown by the electrolyte shows its capability to both transport ions (which is necessary for energy storage functionality) and to withstand applied loads necessary for structural integrity.
- The structural electrode (CF || GF_PEO-SN–NaClO_4_) component was fabricated by pressing the intermediate modulus carbon fibers (possessing hard carbon type microstructures) with the prepared structural electrolyte to boost sodium-ion storage capabilities. It exhibited a considerable tensile strength of 72.3 MPa.
- To investigate sodium-ion insertion capabilities, the structural battery (CF || GF_PEO-SN–NaClO_4_ || Na) was fabricated, and its performance was tested. It provided a typical energy density of 14.2 Wh kg^−1^ at 0.1 C rate, tested for cycling performance, showed good cycling stability, and retained 80% capacity at the end of 200 cycles. The microstructure analysis of the cycled electrodes using scanning electron microscopy revealed small dendrite formation, but the small, uniform Na metal deposition avoided cell short circuits and hence ensured good cycling stability.

The fabricated composites (structural electrolyte and electrode) showed multifunctional characteristics necessary for the design of ambient-temperature sodium-ion structural batteries.

## Figures and Tables

**Table 1:** Table showing the comparison of this work with reported structural electrolyte type and typical energy density values for DOI III type structural batteries.

References	Electrolyte Type	Ion Type	Reported Values		
			C Rate	Specific Capacity (Ah kg^−1^)	Energy Density (Wh kg^−1^)
Liu et al. [10]	Gel-polymer-type electrolyte	Li-ion	0.05C	DNA ^(a)^	35
Meng et al. [35]	Kevlar reinforced gel type electrolyte	Li-ion	DNA ^(a)^	DNA ^(a)^	1.4
Thakur and Dong [36]	Solid polymer-type electrolyte coated on individual carbon fibers	Li-ion	0.5C	23.4	7.6
Moyer et al. [37]	Liquid electrolyte impregnated on Celgard separator	Li-ion	0.1C	30	35
Asp et al. [38]	Bi-continuous phase-type electrolyte with GF plain weave separator	Li-ion	0.05C	8.55	23.6
Asp et al. [38]	Bi-continuous phase-type electrolyte with Whatman GF/A separator	Li-ion	0.05C	4.13	11.6
Siraj et al. [39]	Bi-continuous phase-type electrolyte with GF plain weave separator	Li-ion	0.05C	14.7	41.2
Siraj et al. [39]	Bi-continuous phase-type electrolyte with Whatman GF/A separator	Li-ion	0.05C	9.82	25.9
Iyer et al. [12]	PEO-based all-solid-state composite structural electrolyte with NZSP (Na_3_Zr_2_Si_2_PO_12_) nanoparticle fillers embedded with glass-fiber woven fabric separator	Na-ion	0.1C	10.8	23 *
This work	PEO-based all-solid-state composite structural electrolyte with succinonitrile (C_2_H_4_(CN)_2_) plasticizer fillers embedded with glass-fiber woven fabric separator	Na-ion	0.1C	7.5	14.2 *

* The energy density values mentioned here are for structural battery half-cells (testing of structural cathode with sodium metal anode); ^(a)^ Data not available.

## Data Availability

The original contributions presented in the study are included in the article. Further inquiries can be directed to the corresponding author.

## Supplementary Materials

The following supporting information can be downloaded at: https://www.mdpi.com/article/10.3390/polym16192806/s1, Figure S1: Process chain for the preparation of structural electrolyte; Figure S2: Images showing prepared electrolytes samples; Figure S3: Images of prepared structure electrode samples; Figure S4: Coin Cell assembly for asymmetrical and symmetrical cells; Figure S5: Cell assembly for structural battery half-cell; Figure S6: EIS Nyquist plots of solid polymer electrolytes (SPE) with varying amounts of PEO and SN; Figure S7: Electrochemical impedance spectroscopy (EIS) equivalent circuit for the various electrolytes; Figure S8: Ionic conductivities of electrolytes at different temperatures; Figure S9: Linear sweep voltammetry (LSV) curves of PEO-NaClO_4_ membrane; Figure S10: EIS Nyquist plots of Na || GF_PEO-SN-NaClO_4_ || Na with applied ac perturbation before and after dc polarization; Figure S11: Images from GOM viewer showing the strain measurement on the surface of the structure electrolyte; Figure S12: Images from GOM viewer showing the strain measurement on the surface of the structural electrode; Figure S13: Mean and standard deviation values of tensile strength for the structural electrolyte and structural electrode using five samples; Figure S14: Force–displacement curves of Structural electrolyte (GF_PEO-SN–NaClO_4_) and Structural electrode (CF || GF_PEO-SN–NaClO_4_); Figure S15: Reported cell level energy density and elastic modulus of various structural battery architectures with literature references; Figure S16: Percentage cell capacity retention versus cycle number for the CF || GF_PEO-SN–NaClO_4_ || Na cell; title; Table S1: Table showing the ionic conductivities of various polymer electrolyte compositions at room temperature (25 °C).

## References

[1] European Commission The European Green Deal. https://europa.eu/!DG37Qm.

[2] European Commission Clean Sky Benefits. https://www.clean-aviation.eu/benefits-for-cleaner-greener-healthier-skies.

[3] European Commission Flightpath 2050. https://data.europa.eu/doi/10.2777/15458.

[4] Tesla Battery Pack Architecture. https://futurism.com/take-an-in-depth-look-at-the-tesla-model-3s-new-battery-pack-architecture.

[5] Wetzel E.D. (2004). Reducing Weight: Multifunctional Composites Integrate Power, Communications, and Structure. AMPTIAC Q..

[6] Adam T.J., Liao G., Petersen J., Geier S., Finke B., Wierach P., Kwade A., Wiedemann M. (2018). Multifunctional Composites for Future Energy Storage in Aerospace Structures. Energies.

[7] Thomas J.P., Qidwai W.R., Pogue W.R., Pham G. (2012). Multifunctional structure battery composites for marine systems. J. Compos. Mater..

[8] Pereira T., Guo Z., Nieh S., Arias J., Hahn T. (2008). Embedding thin-film lithium energy cells in structural composites. Compos. Sci. Technol..

[9] Petersen J., Kube A., Geier S., Wierach P. (2022). Structure-Integrated Thin-Film Supercapacitor as a Sensor. Sensors.

[10] Liu P., Sherman E., Jacobsen A. (2009). Design and fabrication of multifunctional structural batteries. J. Power Sources.

[11] Leijonmarck S., Carlson T., Lindbergh G., Asp E.S., Maples H., Bismarck A. (2013). Solid polymer electrolyte coated carbon fibers for structural and novel micro batteries. Compos. Sci. Technol..

[12] Iyer V., Petersen J., Geier S., Wierach P. (2024). Development and multifunctional characterization of a structural sodium-ion battery using a high-tensile-strength poly(ethylene oxide)-based matrix composite. ACS Appl. Energy Mater..

[13] Geier S., Petersen J., Iyer V., Wierach P. Challenges of integrating supercapacitors into structures for space qualification. Proceedings of the 16th European Conference on Spacecraft Structures, Materials and Environmental Testing (ECSSMET 2021).

[14] Asp L.E., Johansson M., Lindbergh G., Xu J., Zenkert D. (2019). Structural battery composites: A review. Funct. Compos. Struct..

[15] Muñoz B.K., Lozano J., Sánchez M., Ureña A. (2024). Hybrid Solid Polymer Electrolytes Based on Epoxy Resins, Ionic Liquid, and Ceramic Nanoparticles for Structural Applications. Polymers.

[16] Jacques E., Kjell M.H., Zenkert D., Lindbergh G., Behm M., Willgert M. (2012). Impact of electrochemical cycling on the tensile properties of carbon fibres for structural lithium-ion composite batteries. Compos. Sci. Technol..

[17] Fredi G., Jeschke S., Boulaoued A., Wallenstein J., Rashidi M., Liu F., Harnden R., Zenkert D., Hagberg J., Lindbergh G. (2018). Graphitic microstructure and performance of carbon fibre Li-ion structural battery electrodes. Multifunct. Mater..

[18] Schneider L.M., Ihrner N., Zenkert D., Johansson M. (2019). Bicontinuous Electrolytes via Thermally Initiated Polymerization for Structural Lithium Ion Batteries. ACS Appl. Energy Mater..

[19] Dong G., Mao Y., Yang G., Li Y., Song S., Xu C., Huang P., Hu N., Fu S. (2021). High-Strength Poly(ethylene oxide) Composite Electrolyte Reinforced with Glass Fiber and Ceramic Electrolyte Simultaneously for Structural Energy Storage. ACS Appl. Energy Mater..

[20] Stevens D.A., Dahn J.R. (2001). The Mechanisms of Lithium and Sodium Insertion in Carbon Materials. J. Electrochem. Soc..

[21] Kjell M.H., Jacques E., Zenkert D., Behm M., Lindbergh G. (2011). PAN-based carbon fiber electrodes for structural lithium-ion batteries. J. Electrochem. Soc..

[22] Zhao Q., Stalin S., Zhao C.Z., Archer L.A. (2020). Designing solid-state electrolytes for safe, energy-dense batteries. Nat. Rev. Mater..

[23] Goikolea E., Palomares V., Wang S., de Larramendi I.R., Guo X., Wang G., Rojo T. (2020). Na-Ion Batteries—Approaching Old and New Challenges. Adv. Energy Mater..

[24] Hayashi A., Noi K., Sakuda A., Tatsumisago M. (2012). Superionic glass-ceramic electrolytes for room-temperature rechargeable sodium batteries. Nat. Commun..

[25] Ma Q.L., Guin M., Naqash S., Tsai C.L., Tietz F., Guillon O. (2016). Scandium-Substituted Na_3_Zr_2_(SiO_4_)_2_(PO_4_) Prepared by a Solution-Assisted Solid-State Reaction Method as Sodium-Ion Conductors. Chem. Mater..

[26] Alarco P.J., Abu-Lebdeh Y., Abouimrane A., Armand M. (2004). The plastic-crystalline phase of succinonitrile as a universal matrix for solid-state ionic conductors. Nat. Mater..

[27] Fan L.Z., Maier J. (2006). Composite effects in poly(ethylene oxide)–succinonitrile based all-solid electrolytes. Electrochem. Commun..

[28] Park C.W., Ryu H.S., Kim K.W., Ahn J.H., Lee J.Y., Ahn H.J. (2007). Discharge properties of all-solid sodium–sulfur battery using poly (ethylene oxide) electrolyte. J. Power Sources.

[29] Yu X., Xue L., Goodenough J.B., Manthiram A. (2020). Ambient-Temperature All-Solid-State Sodium Batteries with a Laminated Composite Electrolyte. Adv. Funct. Mater..

[30] Evans J., Vincent C.A., Bruce P.G. (1987). Electrochemical measurement of transference numbers in polymer electrolytes. Polymer.

[31] Li D., Chen L., Wang T., Fan L.Z. (2018). 3D Fiber-Network-Reinforced Bi-continuous Composite Solid Electrolyte for Dendrite-free Lithium Metal Batteries. ACS Appl. Mater. Interfaces.

[32] Lee B., Paek E., Mitlin D., Lee S.W. (2019). Sodium Metal Anodes: Emerging Solutions to Dendrite Growth. Chem. Rev..

[33] Qiang Z., Chen Y.M., Xia Y.F., Liang W.F., Zhu Y., Vogt B.D. (2017). Ultra-long cycle life, low-cost room temperature sodium-sulfur batteries enabled by highly doped (N,S) nanoporous carbons. NanoEnergy.

[34] Feng M., Wang S., Yu Y., Feng Q., Yang J., Zhang B. (2017). Carboxyl functionalized carbon fibers with preserved tensile strength and electrochemical performance used as anodes of structural lithium-ion batteries. Appl. Surf. Sci..

[35] Meng C., Muralidharan N., Teblum E., Moyer K.E., Nessim G.D., Pint C.L. (2018). Multifunctional Structural Ultrabattery Composite. Nano Lett..

[36] Thakur A., Dong X. (2020). Printing with 3D continuous carbon fiber multifunctional composites via UV-assisted coextrusion deposition. Manuf. Lett..

[37] Moyer K., Meng C., Marshall B., Assal O., Eaves J., Perez D., Karkkainen R., Roberson L., Pint C.L. (2020). Carbon fiber reinforced structural lithium-ion battery composite: Multifunctional power integration for CubeSats. Energy Storage Mater..

[38] Asp L.E., Bouton K., Carlstedt D., Duan S., Harnden R., Johannisson W., Johansen M., Johansson M.K.G., Lindbergh G., Liu F. (2021). A Structural Battery and its Multifunctional Performance. Adv. Energy Sustain. Res..

[39] Siraj M.S., Tasneem S., Carlstedt D., Duan S., Johansen M., Larsson C., Xu J., Liu F., Edgren F., Asp L.E. (2023). Advancing Structural Battery Composites: Robust Manufacturing for Enhanced and Consistent Multifunctional Performance. Adv. Energy Sustain. Res..

[40] Danzi F., Salgado R.M., Oliveira J.E., Arteiro A., Camanho P.P., Braga M.H. (2021). Structural Batteries: A Review. Molecules.

