# Supplementary material for: Design and Characterization of Poly(ethylene oxide)-Based Multifunctional Composites with Succinonitrile Fillers for Ambient-Temperature Structural Sodium-Ion Batteries
Source: Polymers (Basel). 2024 Oct 3;16(19):2806. doi: 10.3390/polym16192806 (PMC11478946; doi:10.3390/polym16192806)
Supplement: Supplementary file 1 [file polymers-16-02806-s001.zip › polymers-3215849-supplementary.pdf]

Supplementary Information (SI)

Design and Characterization of Poly(ethylene oxide)-Based Multifunctional Composites with Succinonitrile Fillers for Ambient-Temperature Structural Sodium-Ion Batter-ies

V. Iyer[1,2,*], J. Petersen[1,2], S. Geier[1,2], and P. Wierach[1,2,3]

[1] *Cluster of Excellence SE2A – Sustainable and Energy-Efficient Aviation, Technische Universität Braunschweig, Braunschweig, 38108, Germany*

[2] *Department of Multifunctional Materials, German Aerospace Center (DLR), Institute of Lightweight Systems, Lilienthalplatz 7, Braunschweig, 38108, Germany.*

[3] *Institute of Polymer Materials and Plastics Engineering, Technische Universität Clausthal, Agricolastrasse 6, Clausthal-Zellerfeld, 38678, Germany*

*E-mail: vasan.iyer@dlr.de

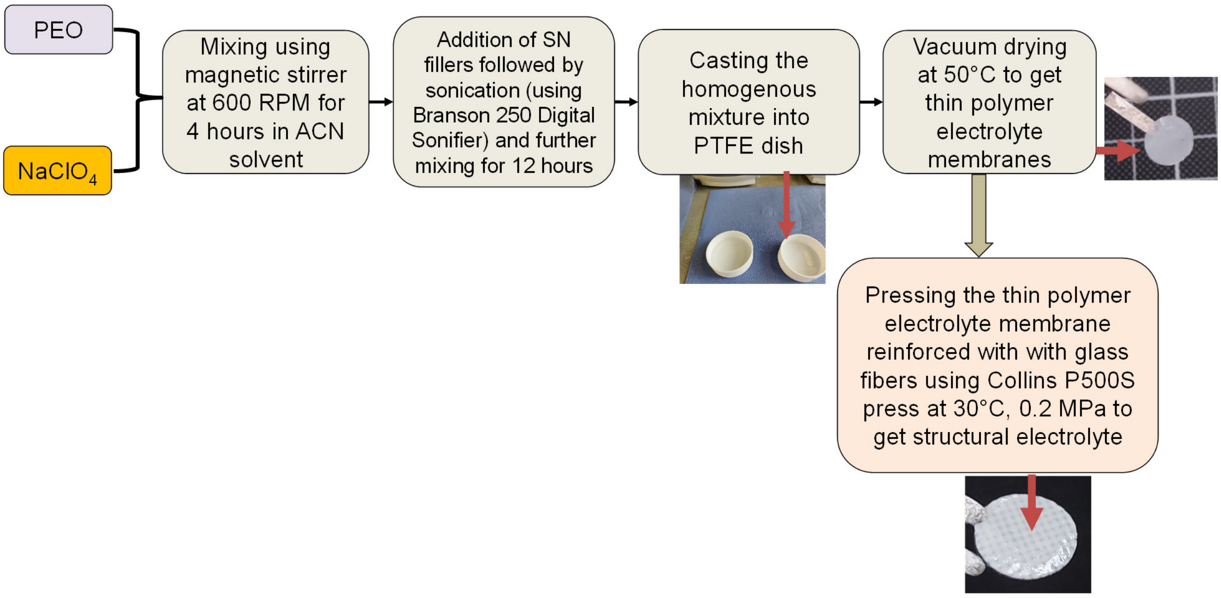

Figure S1: Process chain for the preparation of structural electrolyte.

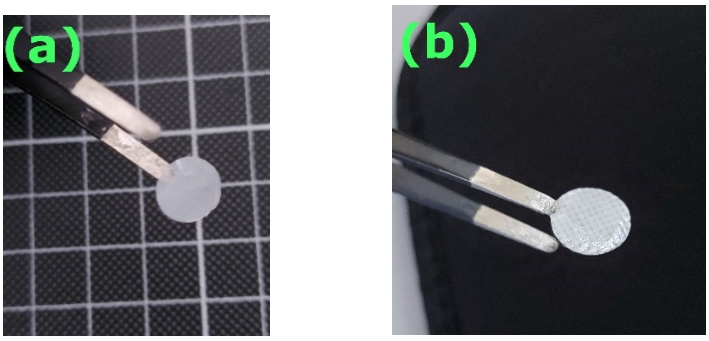

Figure S2. Images showing prepared electrolytes samples: a) Thin polymer membrane PEO-SN-NaClO4. b) Structural electrolyte GF_PEO-SN-NaClO4.

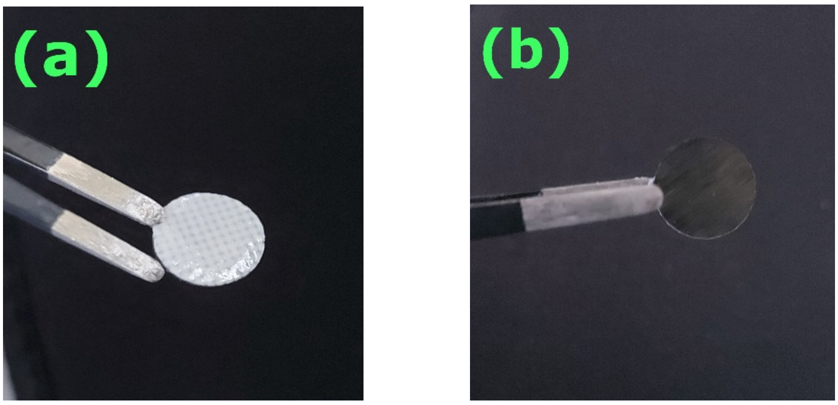

Figure S3. Images of prepared structure electrode samples: a) Upper surface showing polymer membrane PEO-SN-NaClO4 reinforced with glass fiber. b) Lower surface showing carbon fiber electrode laminated onto the structural electrolyte.

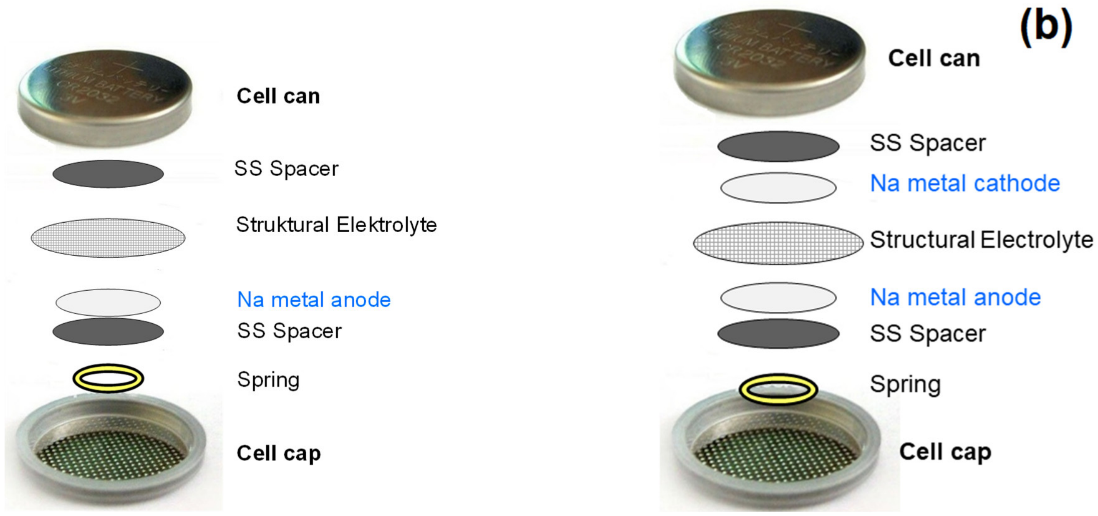

Figure S4: Coin Cell assembly for: **(a)** Asymmetrical cells. **(b)** Symmetrical cells..

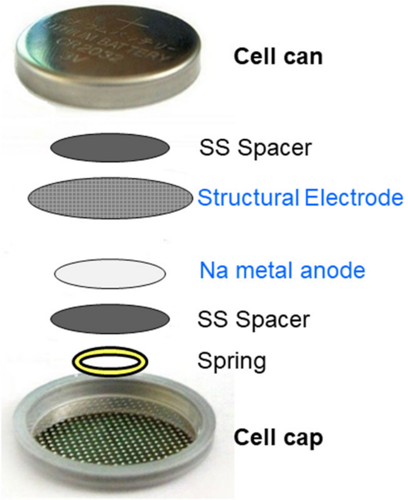

Figure S5: Cell assembly for structural battery half-cell.

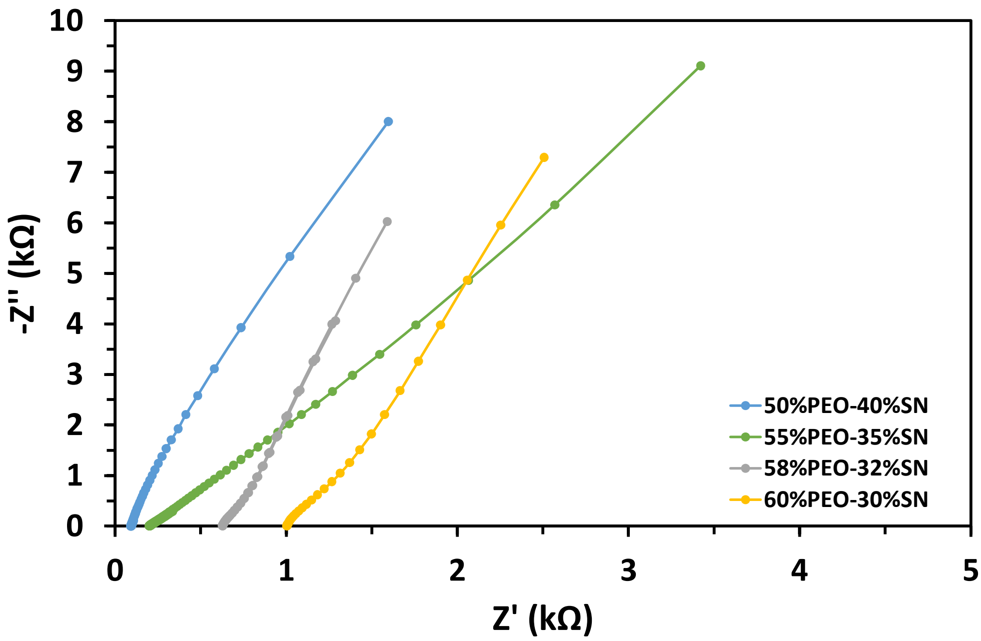

Figure S6: EIS Nyquist plots of solid polymer electrolytes (SPE) with varying amounts of PEO and SN.

Table S1: Table showing the ionic conductivities of various polymer electrolyte compositions at room temperature (25°C)

Electrolyte composition	Ionic conductivity
50% PEO-40% SN-10% NaClO4	0.000309625
55% PEO-35% SN-10% NaClO4	0.000132696
58% PEO-32% SN-10% NaClO4	4.61553E-05
60% PEO-30% SN-10% NaClO4	3.18471E-05

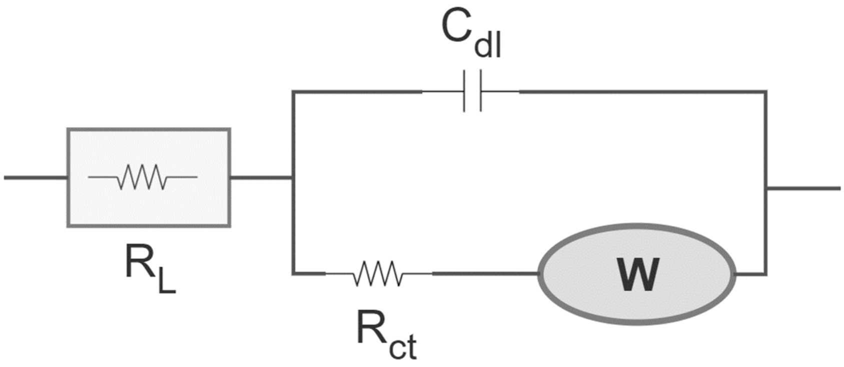

Figure S7. Electrochemical impedance spectroscopy (EIS) equivalent circuit for the various electrolytes, where R_L is the bulk resistance, R_{ct} is the interface or charge-transfer resistance, C_{dl} is the double layer interface capacitance, and W – Warburg element represent the solid-state diffusion of Na^+ into the electrodes.

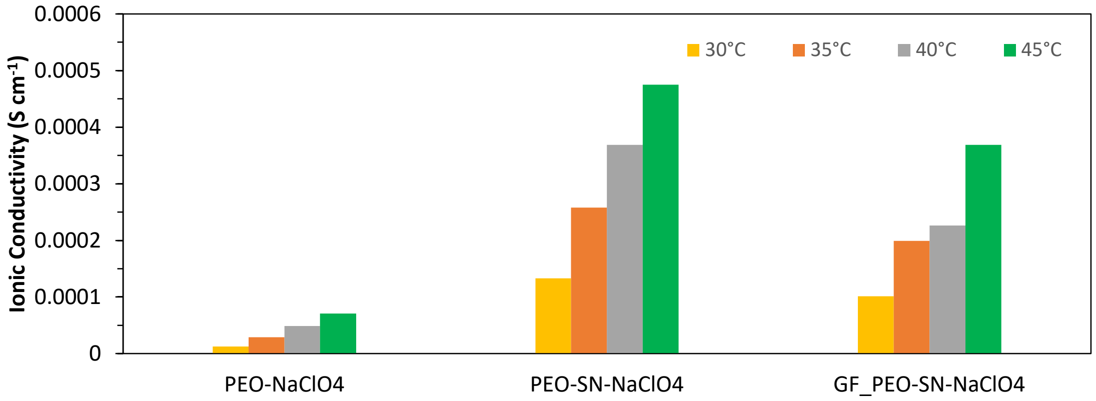

Figure S8. Ionic conductivities of electrolytes at different temperatures

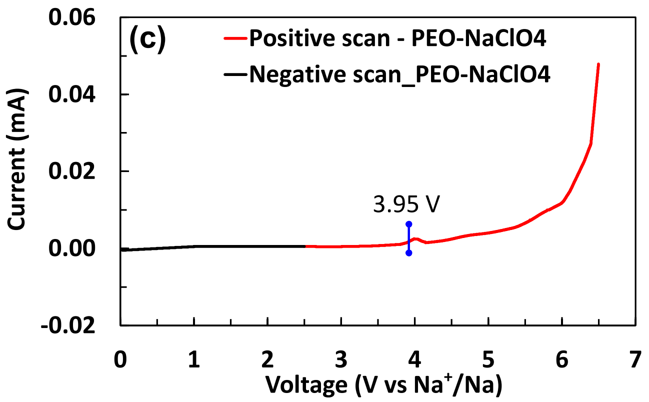

Figure S9. Linear sweep voltammetry (LSV) curves of PEO-NaClO4 membrane [1].

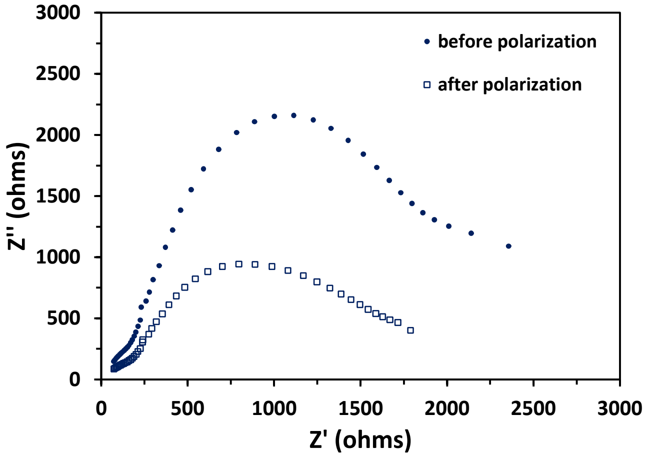

Figure S10. EIS Nyquist plots of Na || GF_PEO-SN-NaClO4 || Na with applied ac perturbation before and after dc polarization.

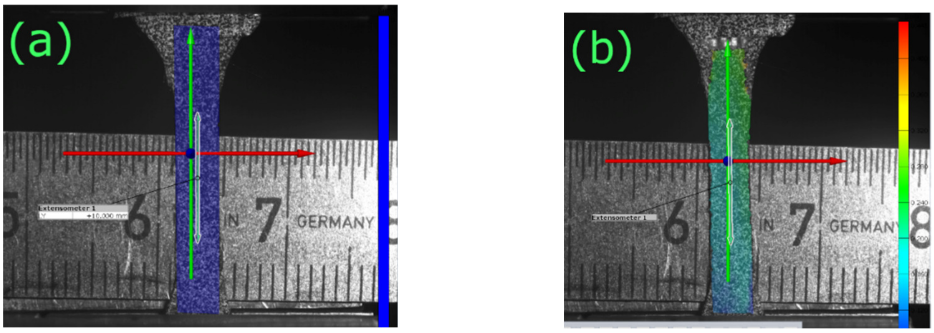

Figure S11. Images from GOM viewer showing the strain measurement on the surface of the structure electrolyte GF_PEO – SN – $NaClO_4$: (a) Starting point of tensile test. (b) structural electrolyte sample during tensile test showing elongation in the extensometer.

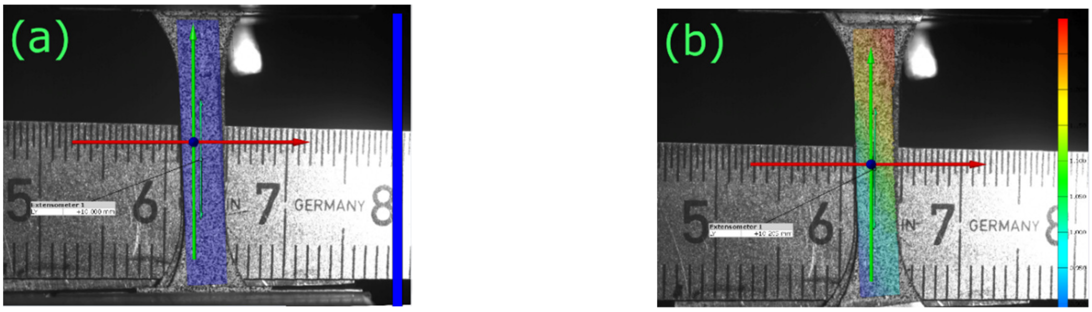

Figure S12. a) Images from GOM viewer showing the strain measurement on the surface of the structural electrode CF||GF_PEO – SN – $NaClO_4$: (a) Starting point of tensile test. (b) Structural electrode sample during tensile test showing elongation in the extensometer.

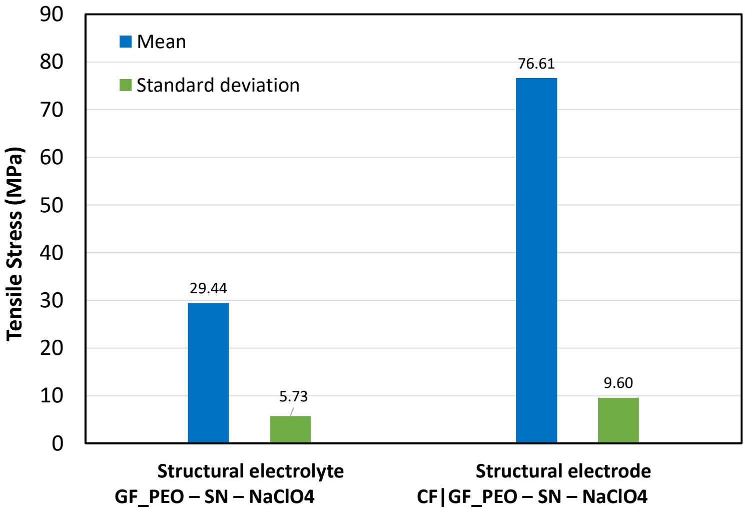

Figure S13. Mean and standard deviation values of tensile strength for the structural electrolyte and structural electrode using five samples.

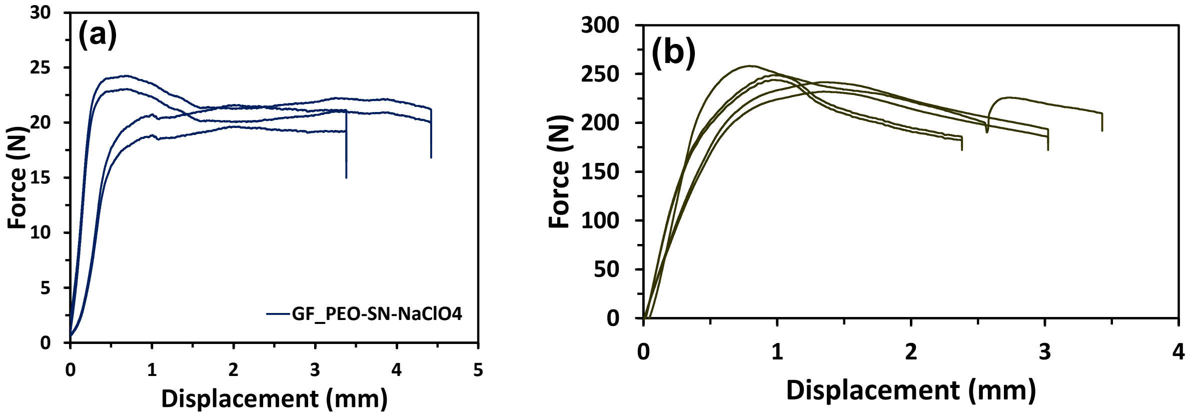

Figure S14. Force – displacement curves: **(a)** Structural electrolyte GF_PEO-SN-$NaClO_4$. **(b)** Structural electrode CF || GF_PEO-SN-$NaClO_4$.

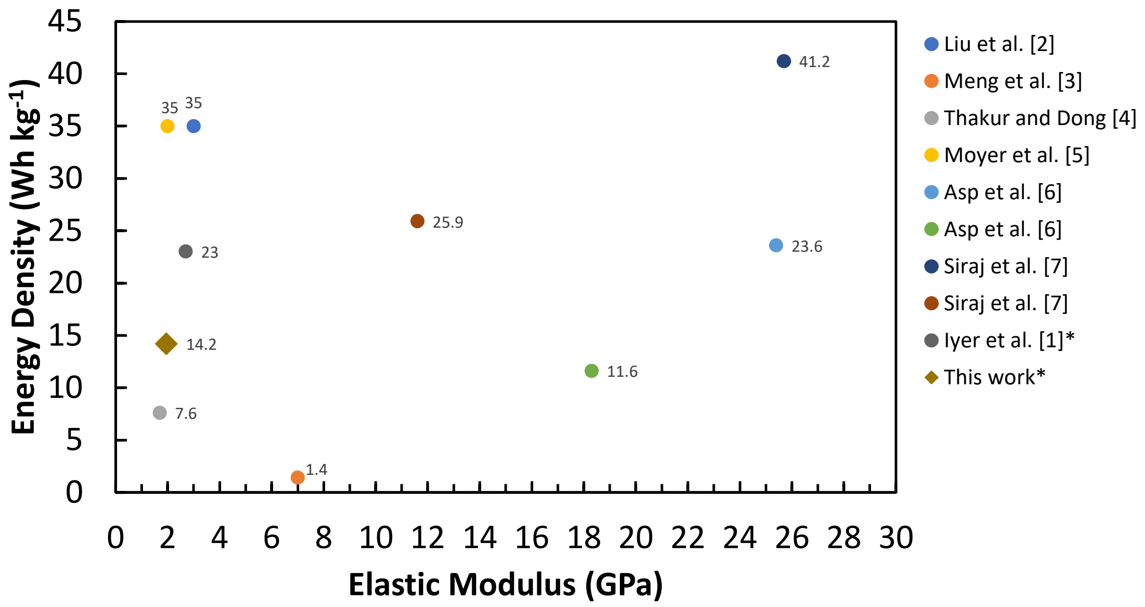

(*To be noted, these work reports structural battery half-cell energy density values (testing of structural cathode with sodium metal anode) and not full structural battery)

Figure S15. Reported cell level energy density and elastic modulus of various structural battery architectures with literature references (energy density values highlighted in plot).

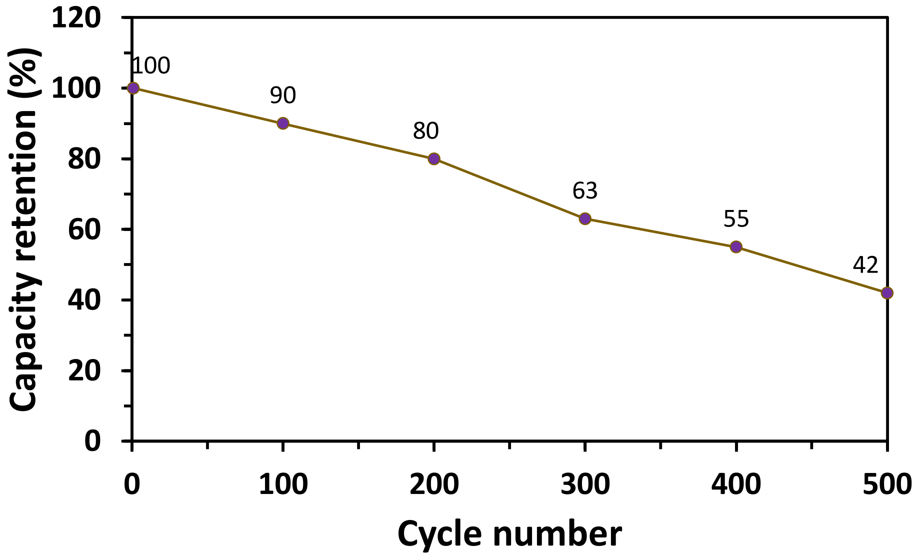

Figure S16. Percentage cell capacity retention versus cycle number for the CF || GF_PEO-SN-$NaClO_4$ || Na cell.

References

[1] V. Iyer, J. Petersen, S. Geier, P. Wierach, Development and multifunctional characterization of a structural sodium-ion battery using a high-tensile-strength poly(ethylene oxide)-based matrix composite, ACS Appl Energy Mater. 7 (2024), 3968-3982, https://doi.org/10.1021/acsaem.4c00281.

[2] P. Liu, E. Sherman, A. Jacobsen, Design and fabrication of multifunctional structural batteries, *Journal of Power Sources* 189 (2009), 646–650, https://doi.org/10.1016/j.jpowsour.2008.09.082.

[3] C. Meng, N. Muralidharan, E. Teblum, K. E. Moyer, G. D. Nessim, C. L. Pint, Multifunctional structural ultrabattery composite, *Nano Lett.* 18 (2018), 7761-7768, https://doi.org/10.1021/acs.nanolett.8b03510.

[4] A. Thakur, X. Dong, Printing with 3D continuous carbon fiber multifunctional composites via UV-assisted coextrusion deposition, *Manuf. Lett.* 24(2020), 1-5, https://doi.org/10.1016/j.mfglet.2020.02.001.

[5] K. Moyer, C. Meng, B. Marshall, O. Assal, J. Eaves, D. Perez, R. Karkkainen, L. Roberson, C. L. Pint, Carbon fiber reinforced structural lithium-ion battery composite: Multifunctional power integration for CubeSats, *Energy Stor. Mater.* 24(2020), 676-681, https://doi.org/10.1016/j.ensm.2019.08.003.

[6] L. E. Asp, K. Bouton, D. Carlstedt, S. Duan, R. Harnden, W. Johannisson, M. Johansen, M. K. G. Johansson, G. Lindbergh, F. Liu, K. Peuvot, L. M. Schneider, J. Xu, D. Zenkert, A structural battery and its multifunctional performance. *Adv. Energy Sustainability Res.* 2(2021), 2000093, https://doi.org/10.1002/aesr.202000093.

[7] M. S. Siraj, S. Tasneem, D. Carlstedt, S. Duan, M. Johansen, C. Larsson, J. Xu, F. Liu, F. Edgren, L. E. Asp, Advancing structural battery composites: Robust manufacturing for enhanced and consistent multifunctional performance *Adv. Energy Sustainability Res.* 4(2023), 2300109, https://doi.org/10.1002/aesr.202300109.
